# Genomic, transcriptomic and epigenomic signatures of ageing and cold adaptation in the Antarctic clam *Laternula elliptica*

**DOI:** 10.1098/rsob.250009

**Published:** 2025-05-21

**Authors:** Victoria A. Sleight, Melody S. Clark, Meghan K. Yap-Chiongco, Frances Turner, Kevin M. Kocot

**Affiliations:** ^1^ School of Biological Sciences, University of Aberdeen, Aberdeen, UK; ^2^ British Antarctic Survey, Cambridge, UK; ^3^ Department of Biology, University of Copenhagen, Copenhagen, Denmark; ^4^ Department of Biological Sciences, University of Alabama, Tuscaloosa, AL, USA; ^5^ Edinburgh Genomics (Genome Science), University of Edinburgh, Edinburgh, UK; ^6^ Alabama Museum of Natural History, University of Alabama, Tuscaloosa, AL, USA

**Keywords:** Anomalodesmata, benthic, shell repair, life-history trait, biomineralization, immune function

## Abstract

Genomic data are lacking for most Antarctic marine invertebrates, predicating our ability to understand physiological adaptation and specific life-history traits, such as longevity. The environmental stress response of the Antarctic infaunal clam *Laternula elliptica* is much diminished in older adult animals compared with younger juvenile individuals. However, the mechanism underlying this reduced capacity is unknown. In this study, we describe and analyse the genome of *L. elliptica* and use it as a tool to understand transcriptomic responses to shell damage across different age cohorts. Gene expression data were combined with reduced representation enzymic methyl sequencing to identify if methylation was acting as an epigenetic mechanism driving age-dependent transcriptional profiles. Our transcriptomic results demonstrated a clear bipartite molecular response in *L. elliptica*, associated with a rapid growth phase in juveniles and a stabilization phase in reproductively mature adults. Genes active in the response to damage repair in juvenile animals are silent in adults but can be reactivated after several months following damage stimulus; however, these genes were not methylated. Hence, the trigger for this critical and imprinted change in physiological state is, as yet, unknown. While epigenetics is likely involved in this process, the mechanism is unlikely to be methylation.

**Figure:**
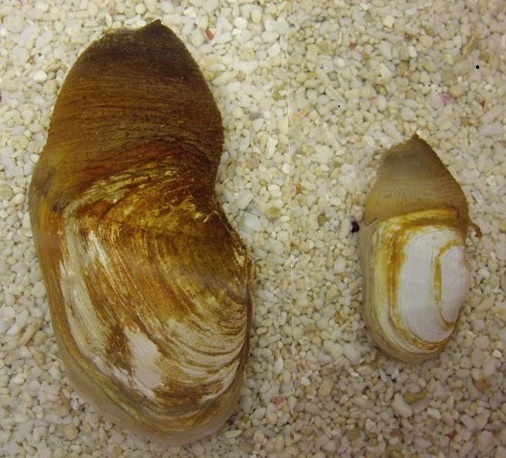

## 1. Background

Many species, particularly those in colder regions, can be very long-lived with age-dependent responses to climate change, and other stressors significantly impacting future recruitment and sustainability of populations [1,2]. Therefore, understanding age-dependent responses and whether these are hardwired into genomes is critical for predicting future changes in marine biodiversity in cold regions. To date, experimental studies describing and predicting marine species’ resilience to climate change have used either average-sized adults with a narrow size range (to minimize experimental variation) or embryos and early-stage larvae [1,3]. Bivalve molluscs are an excellent group to study the effects of longevity on physiology in natural populations, as growth rate and age data are recorded in shells, and they inhabit a diversity of gradients in physical parameters [4]. To date, research on ageing and life-history traits in bivalves has largely focused on extrinsic factors (temperature, metabolism, caloric intake and predation), although there has been a more recent focus on intrinsic factors (genetics) with the increasing availability of genomes and large transcriptome datasets [5–7]. Despite knowledge gained from these studies, the genetic and molecular mechanisms underpinning how longevity impacts life-history traits and how such processes are controlled remain largely unexplained.

A relatively well-studied long-lived bivalve mollusc is the Antarctic anomalodesmatan clam *Laternula elliptica* (King & Broderip, 1831). This species can be highly abundant in the region and has a circumpolar distribution [8]. It is the largest infaunal mollusc in the Southern Ocean, playing a major role in benthopelagic coupling [9,10]. It can live to approximately 36 years, with the shells of older animals measuring up to 100 mm [11]. This species has long been proposed as a model for ageing studies, particularly within a marine ecology context [12]. Whole-organism physiological studies have shown that older animals bury less rapidly, are more thermally sensitive and are more affected by sedimentation and injury compared with younger animals [13–16]. In addition, transcriptomic analyses of environmental responses in *L. elliptica* have also shown age-specific profiles and a lower level of cellular response in older animals [2,17,18]. However, understanding of the underlying cellular mechanisms underpinning these age-dependent responses is currently limited. Some studies have indicated that mitochondrial energetics, reactive oxygen species (ROS) and DNA damage play a substantial role in molluscan ageing processes [4,19], while recent molecular studies using *Argopecten* scallops have demonstrated a regulatory role for the PI3K/Akt/FoxO pathway, which is involved in cell proliferation and apoptosis [6,20].

How ageing and related pathways are controlled through life history across molluscs, and indeed, the tree of life is unclear; one hypothesis is that epigenetic factors may be key regulators [21]. DNA methylation is an epigenetic modification that plays a crucial role in regulating gene expression. It involves the addition of a methyl group to the DNA molecule, typically at cytosine residues within CpG dinucleotides and is associated with gene silencing and transcriptional repression. Recent studies have shown that DNA methylation patterns can be affected by environmental factors and can contribute to phenotypic differences among individuals, including age-dependent changes in gene expression [21,22]. Thus, understanding DNA methylation patterns, alongside transcriptomic studies, in long-lived species such as *L. elliptica* could provide valuable insights into the underlying mechanisms of ageing and the potential role of epigenetic modifications in regulating age-dependent life-history traits and responses to stress.

**Figure 1.**
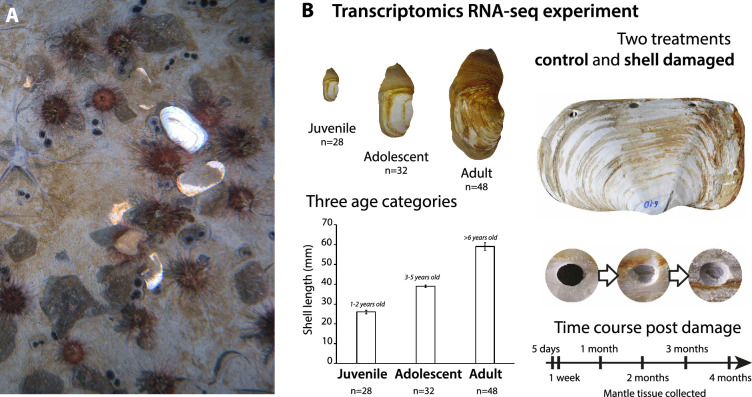
*Laternula elliptica* and experimental design of transcriptome experiment. (A) Photo of benthic community on Antarctic seabed, in frame are buried *L. elliptica* with siphon holes (paired circular structures protruding from the sediment) visible in sea floor mud, alongside *L. elliptica* shells that are the remains from previously damaged animals. (B) Experimental design of *L. elliptica* transcriptomics experiment with three different age classes (juvenile, adolescent and adult) and time course of damage repair sampling as drilled hole gradually occluded.

Here, we have assembled and annotated a draft genome for *L. elliptica* and conducted transcriptomic and methylation analyses across life-history stages (figure 1). Our working hypothesis for the age-dependent shell damage-repair response studied here was that the more active transcriptional profile (and rapid shell repair) of juvenile *L. elliptica* diminishes with age due to methylation of genes only expressed in small pre-reproductive animals, which take a long time to reactivate in adult animals with more extended shell repair times.

## 2. Material and methods

### 2.1. Animal collection and sampling

All *L. elliptica* specimens for these experiments were collected by SCUBA divers from Hangar Cove near Rothera Research Station, Adelaide Island on the Antarctic Peninsula (67° 34′ 07″ S, 68° 07′ 30″ W) at depths of 10−15 m. ***Sampling for genome and methylation experiment:*** Ten animals were sampled comprising two groups: one group of juveniles (pre-reproductive) (*n* = 5) and the other of adult animals (*n* = 5). Animals were initially chosen by size and then aged by visual inspection of annual growth rings in their shells [23]. Juvenile animals had a mean shell length of 30.32 mm (± 2.97 mm s.e.; range 20.80– 36.65 mm) and a mean age of 4.8 years (±0.49 s.e.; range 4−6 years). Adult animals had a mean shell length of 58.88 mm (± 5.89 s.e.; range 48.50–81.50 mm) and a mean age of 9.2 years (±0.80 s.e.; range 8–12 years). The two age cohorts were significantly different in age (*p* = 0.003, *t*-value −4.69, d.f. = 6). Animals were maintained in the flow-through aquarium at the laboratory at Rothera Research Station before transferring to the BAS Cambridge aquarium, where they were sacrificed. Pieces of mantle tissue were dissected from the body with sterile tools, flash frozen in liquid nitrogen and stored at −80°C. The individual used for genome sequencing was designated Le2, with an estimated age of 13−14 years (i.e. an adult animal) and has been registered with the Alabama Museum of Natural History with accession number 23548 (https://arctos.database.museum/guid/ALMNH:Inv:23548).

### 2.2. Genome assembly

***DNA extraction:*** Frozen mantle tissue from individual Le2 was sent on dry ice to the University of California Davis DNA Technologies Core Facility for DNA extraction using a Nanobind Tissue Big DNA Kit (Circulomics, Baltimore, MD) following the manufacturer’s protocol. Extracted DNA was cleaned with equal volumes of phenol/chloroform using phase-lock gels (Quantabio, Beverly, MA) and precipitated by adding 0.4× volume of 5 M ammonium acetate and 3× volume of ice-cold 100% ethanol. The DNA pellet was then washed twice with 70% ethanol and resuspended in an elution buffer (10 mM Tris, pH 8.0). The purity of the DNA was accessed using a NanoDrop spectrophotometer (260:280 and 260:230 ratios), and the integrity of the high-molecular-weight (HMW) gDNA was verified on a Femto pulse system (Agilent Technologies, Santa Clara, CA). ***Genome sequencing:*** A PacBio HiFi SMRTbell library was constructed using the SMRTbell Express Template Prep Kit v2.0 (Pacific Biosciences, Menlo Park, CA) according to the manufacturer’s instructions. HMW gDNA was sheared to a target DNA size distribution between 15 and 20 kb using a Megaruptor (Diagenode, Belgium). Sheared gDNA was concentrated using 0.5× AMPure PB beads (Pacific Biosciences) for the removal of single-strand overhangs at 37°C for 15 min, followed by further enzymatic steps of DNA damage repair at 37°C for 30 min, end repair and A-tailing at 20°C for 10 min and 65°C for 30 min, ligation of v3 overhang adapters at 20°C for 60 min and 65°C for 10 min to inactivate the ligase, then nuclease treated at 37°C for 1 h. The SMRTbell library was purified and concentrated with 0.8× Ampure PB beads for size selection using a BluePippin to collect fragments >15 kb. The resulting library was sequenced at UC Davis DNA Technologies Core (Davis, CA) using one SMRT Cell 8M Tray with Sequel II sequencing chemistry v. 2.0 and a 30 h movie on a PacBio Sequel II sequencer. ***Genome assembly:*** Resulting circular consensus sequences (CCS) were assembled using hifiasm 0.13-r308 [24] with an intermediate level of redundant haplotype assembly purging (‘-l 2’) with the integrated implementation of purge_dups [25] on the University of Alabama High-Performance Computing cluster. Assembly quality was assessed with QUAST 5.0.2 [26] and completeness with BUSCO 4.0.2 [27] using the Metazoa odb_10 dataset and the ‘--mode genome’ and ‘--long’ options. Genome size and heterozygosity were independently estimated based on CCS reads using GenomeScope 2 [28] with a k-mer of 21. ***Genome annotation:*** For structural annotation, repeats in the final contamination-filtered assembly were annotated and softmasked with RepeatMasker 4.1.2 (http://www.repeatmasker.org) using a custom repeat database generated with RepeatModeler 2.0.1 [29]. For RepeatMasker, the ‘gc’ value was set to 38 based on the GC-content reported by QUAST. The engine used for both programs was rmblast (http://www.repeatmasker.org/rmblast/). Available *L. elliptica* transcriptome data (NCBI SRA accession numbers SRR1713116, SRR1713117, SRR1713118, SRR1713176, SRR1713179, SRR1713180, SRR1713181, SRR1691077, SRR1691087, SRR1691246, SRR1691261, SRR1691274, SRR1691282, SRR1687084, SRR1687177, SRR1687181, SRR1687224, SRR1687225 and SRR1687226) were downloaded from NCBI. We performed quality and adapter trimming and filtering on the transcriptome reads using TrimGalore 0.6.10 (https://github.com/FelixKrueger/TrimGalore) [30] with the following settings: ‘-q 30 --illumina --length 50 --trim-n’. The trimmed and filtered transcriptome reads were then mapped to the genome using STAR 2.4 v. 2.4.0 k (https://github.com/alexdobin/STAR) [31] with the following settings: ‘--chimSegmentMin 50 --outFilterType BySJout’. Annotation of protein-coding genes was performed with BRAKER 2.1.6 [32] using the output of STAR as a training data, and evidence with the settings ‘--softmasking --crf’ was used. For functional annotation of the predicted proteins, InterProScan 5.36−75.0 [33] was used with the ‘-dp -iprlookup -goterms -pa -b’ settings.

### 2.3. Comparative genomics analyses

For comparative analyses, publicly available bivalve genomes spanning the diversity of Euheterodonta plus outgroups from Unionida and Pteriomorpha were downloaded. Genomes lacking publicly available annotations were annotated in BRAKER as described above except that protein evidence compiled from the TransDecoder-translated transcriptomes of 24 diverse bivalves was provided as evidence for gene modelling (electronic supplementary material, S1). BUSCO was run on the predicted proteomes as described above except that the ‘--mode protein’ option was used. Only genomes whose predicted proteomes had a BUSCO completeness score >80% were retained for comparative analyses (24 taxa including *L. elliptica* (electronic supplementary material, S1). We used OrthoFinder 2.4.0 [34] with an inflation parameter of 2.1 to identify homologous amino acid sequences among taxa. The resulting groups were filtered to identify strictly orthologous sequences following a bioinformatic pipeline routinely used in the Kocot laboratory [35]. Briefly, we deleted short sequences <100 amino acids and then retained only groups sampled for ≥75% of taxa. These were aligned with MAFFT 7.310 [36], putatively mistranslated regions removed with HmmCleaner 0.180750 [37] and the resulting alignments were trimmed to remove ambiguously aligned regions with BMGE 1.12.2 [38]. Approximately maximum likelihood (ML) trees were constructed for each alignment with FastTree 2 [39], and PhyloPyPruner 0.9.5 (https://pypi.org/project/phylopypruner) was used to identify strictly orthologous sequences sampled for ≥75% of taxa. The resulting alignments were concatenated, and the resulting supermatrix was partitioned by gene and analysed using ML in IQ-TREE 2 [40] using the best-fitting model for each partition (-m MFP) and 1000 rapid bootstrap replicates. CAFE5 [41] was used to examine gene expansions and reductions. The phylogram of the ML tree inferred with IQ-TREE 2 was converted into an ultrametric tree in r8s v. 1.81 [42]. The input for r8s was produced using the CAFE5 script prep_r8s.py using the following parameters: -i bivalve.txt -o r8s_ctl_file.txt -s 2808205 p ‘Chlamys_farreri’, ‘Laternula_elliptica’ -c 497. Bivalve.txt represents the rooted tree produced from IQ-TREE2, while -c and -p represent the calibration points of 497 million years ago between *Chlamys farreri* and *L. elliptica* (after [43,44], as explained in [45]). The resulting file was used as input for r8s using default parameters. Orthogroup (=gene family) counts for each species were obtained using the output file from OrthoFinder Orthogroups.GeneCount.tsv. Gene families with a large gene copy number variance (>100) were removed using the CAFE5 script clade_and_size_filter.py with default parameters. For functional annotation of orthogroups, InterProScan 5.360−92.0 [33] was used with the ‘-iprlookup -goterms -appl Pfam -b’ settings. First, CAFE5 was run using the ultrametric tree and filtered gene counts to estimate an error model to account for errors in sequencing, coverage differences and clustering with the parameters -p -e. CAFE5 was then run again with the same parameters using the error model (-eBase_error_model.txt) to estimate the birth–death parameter *λ* (probability that any gene will be gained or lost) [46]. The output of CAFE5 was then visualized using CafePlotter 0.2.0 (https://github.com/moshi4/CafePlotter). Orthogroup expansion and contraction were further investigated across the tree and specifically in *L. elliptica* using the result_summary.tsv file from CafePlotter and the output of InterProScan.

### 2.4. Transcriptomics

***Sampling for RNA-Seq:*** A time course experiment to examine biomineralization pathways was conducted on three age categories of *L. elliptica* (juvenile = 1−2 years, adolescent = 3−5 years and adults were >6 years) as previously described in [47] (figure 1). ***RNA extraction and sequencing:*** Four individuals were sampled in each category (time × treatment), with the three highest quality RNA samples per category sent for library preparation. Total RNA was extracted from tissue samples using Tri-Reagent (Sigma-Aldrich, UK) according to the manufacturer’s instructions with further purification performed using the RNeasy clean-up kit (Qiagen), which included a DNase step. All RNA samples were analysed for concentration and quality by a spectrophotometer (NanoDrop, ND-1000) and on an Agilent 2200 TapeStation. cDNA libraries were made for each individual in each of the age experiments (*n* = 78). Library preparation was conducted by the Earlham Institute, Norwich, UK. Stranded libraries were prepared using a NEXTflex™ Rapid Illumina Directional RNA-Seq Library Prep Kit, and sequencing was carried out over 5 lanes on a Hi-Seq 2000 generating 125 base paired-end reads. ***Bioinformatics analyses:*** RNAseq data were downloaded from NCBI SRA accession [47] and aligned to the genome using HISAT2 (cite) with the options ‘-q --met-stderr --known-splicesite-infile splice_sites.txt’. The hisat2_extract_splice_sites.py python script bundled with HISAT2 was used to extract splice sites from the gene models predicted by BRAKER and generate splice_sites.txt. Expression levels were quantified at the gene level using edgeR. Differential expression analysis was conducted using a negative binomial additive general linear model with a quasi-likelihood *F*-test. The *p*-values were adjusted for multiple testing using the Benjamini–Hochberg method to control the false discovery rate (FDR), cut-offs for statistical significance were used (FDR < 0.05). Differentially expressed genes were putatively annotated based on sequence similarity searched using blastx against Uniprot (http://www.uniprot.org/), the RefSeq non-redundant database (https://www.ncbi.nlm.nih.gov/refseq/about/nonredundantproteins/) and using InterPro scan to identify pfam domains. Differentially expressed genes were screened for functional categories relating to ageing and tested for functional enrichment using String DB (https://string-db.org/). Differential expression and enrichment results and annotations are available for download. We identified the 100 most highly expressed genes in juvenile control animals that were expressed to a lesser extent in control adolescents and not expressed in control adults (electronic supplementary material, S2). In this analysis, the top 100 most significant genes, i.e. most significantly upregulated in juvenile animals compared with adults, were identified and the results ranked on FDR. The genes with the top 100 smallest FDR values were taken forward for further analyses. There was no FC cut-off, but the FDR of the most significant gene was 0.0000000219 and the least (out of 100) significant was 0.0114. The respective logFC values were largest = 6.72 and smallest = 2.39. To identify what happens to these top 100 juvenile genes during damage perturbation, we conducted a full differential gene expression (DGE) between control and damaged in adult animals and then filtered the results to discover if any of the ‘top 100 juvenile genes’ were significantly upregulated during damage.

### 2.5. Methylation study

***DNA extraction and sequencing:*** DNA was extracted from 10 animals using a standard phenol–chloroform protocol, with quantity and quality evaluated by spectrophotometry (NanoDrop, ND-1000) and an Agilent 2200 TapeStation. DNAs were prepared for methylation evaluation using a NEBNext^®^ Enzymatic Methyl-seq kit (New England Biolabs (NEB)) according to the manufacturer’s instructions based on their reduced NEBNext v1.4 Representation EM-seq Beta protocol, up to and inclusive of the adaptor ligation step. The enzyme used for digestion was Msp1 (NEB). The control (pool of all samples—unconverted) was treated differently at the adaptor ligation step, using reagents from an NEB Ultra II kit and following the associated protocol. All samples were cleaned up with size selection (240−290 bp), with duplicates of each sample combined for the clean-up and size selection, following the Beta protocol. The experimental samples were then treated according to the Beta protocol for the oxidation of 5mC/5hmC and sample purification, with control samples left at −20°C. The deamination of cytosines followed the formamide denaturation protocol. The experimental samples were then processed through to library amplification according to the Beta protocol with eight polymerase chain reaction (PCR) cycles. A single control sample was created by pooling the control samples from each individual. The control sample was processed using the Ultra II protocol (NEB) with eight PCR cycles. All samples were purified using the NEBNext sample purification beads. Libraries were quality-controlled, pooled and sequenced on 1 lane of a NovaSeq SP 300 cycle flowcell at 150PE with 5% PhiX. ***Bioinformatics analyses:*** Default values were used for all parameters in all software used unless otherwise stated. ***Data pre-processing:*** Reads were trimmed using Cutadapt (v. 3.5.1). Reads were trimmed for quality at the 3′ end using a quality threshold of 30 and for the TruSeq adapter sequence (AGATCGGAAGAGC). Reads after trimming were required to have a minimum length of 35. ***Identification of polymorphic sites:*** As converted bases appear as mismatches between ‘T’s in the sequenced data and ‘C’s in the reference, C->T single-nucleotide polymorphisms can be mistaken for unmethylated bases. Therefore, methylation at polymorphic sites cannot be reliably called, so potentially polymorphic sites were removed before methylation analysis. The polymorphic sites were identified as follows. Reads from the unconverted control sample were aligned to the reference genome (hifiasm.asm.p_ctg.fasta.masked) using bwa mem2 (v. 0.7.17) using the ‘-M’ flag to mark split hits and the ‘-R’ flag to add read groups. This produced a single binary alignment map (BAM) file. The ‘HaplotypeCaller’ tool from the Genome Analysis Tool Kit (GATK)3 (v. 4.2.3.0) was used to call variants i from this BAM file using the ‘ –ploidy 10’ parameter, producing a variant call format (vcf) file. ***Methylation calling*:** Reads were aligned to the reference genome using bwameth2 (v. 0.7.17) using the ‘-M’ flag to mark split hits, and the ‘-R’ flag to add read groups. This produced a single BAM file per sample. Average methylation levels at the ends of reads often differ from the methylation levels within reads due to biases in methylation. MethylDackel4 (v. 0.6.1) was run with ‘mbias’ command to produce methylation bias plots for each sample. Manual inspection of these plots showed variations in methylation levels and 5′ and 3′ ends of the reads, and this was used to inform the ‘–OB’ and ‘–OT’ parameters used by MethylDackel ‘extract’ command in the next step. MethylDackel ‘extract’ command was used to extract methylation calls at CpG sites covered by at least 5 reads, discarding reads with a mapping quality phred score of less than 20, with the parameters: ‘ –OB 6,141,5,0 –OT 10,0,0,154 –maxVariantFrac 0.2 –mergeContext –minDepth 5 -q 20 –minOppositeDepth 1’. One ‘bedGraph’ file per sample was produced. ***Filtering of polymorphic sites:*** The ‘subtractBed’ tool from bedtools5 (v. 2.29.1) was used to remove sites present in the vcf file produced in the variant calling step from the bedGraph files produced in the extraction of methylation calls step. ***Generation of intron and exon only data:*** The ‘intersectBed’ tool from bedtools was used to compare the filtered bedGraph files of methylation data produced in the previous step, with the locations of intron and exons in the genome, to produce intron and exon only data for each sample. ***Calculation of average methylation levels:*** In order to include only the more reliably assessed CpGs, those with total coverage of no more than 20 were excluded. For each sample, the total number of reads showing methylation were divided by the total number of reads to give the mean methylation levels. ***Clustering of CpGs:*** Script ‘combine_CpG_sites.py’ from DMRfinderr6 (v. 0.3) was used to combine the separate bedGraph files from each sample to generate a file of combined methylation calls for genomic regions. Default parameters were used to create regions that were no more the 500 bp long and contained at least three CpGs, no more than 100 bp apart. This was performed separately for the whole genome data, the exon only data and the intron only data. ***Identification of differentially methylated regions:*** Script ‘merge_sums_for_dmr.R’ from ‘DMRfinder’ was run with parameters to ‘-p 1 -d 0’ assess differential methylation in all CpG regions identified in the previous step. This was performed separately for the whole genome data, the exon only data and the intron only data. In order to limit the analysis to those regions with sufficient data to make meaningful comparisons between adult and juvenile samples, the regions were filtered to remove those not covered by more than 20 reads in at least 2 adult and at least 2 juvenile samples. The FDR of differential methylation was calculated for the remaining regions using the ‘p.adjust’ function in R.

## 3. Results

We assembled and annotated a highly contiguous and complete draft genome for the Antarctic clam *L. elliptica* (King & Broderip, 1831), which is the first representative of the bivalve clade Anomalodesmata sequenced to date. This genome and its annotation have provided data to understand the evolution of this species and a mapping scaffold for transcriptomic and methylation data. Transcriptome and methylation data were used to identify age-specific cellular processes and the potential age-specific impact of methylation (one of the mechanisms underpinning epigenetic effects) on gene expression.

### 3.1. Genome description: contiguity, annotation and bivalve phylogeny

HiFi sequencing on one PacBio Sequel II SMRT Cell yielded 1 389 345 reads with an average CCS read length of 16 172 bp totalling 22.467 Gbp. GenomeScope analysis inferred a genome size of 727 Mbp and a heterozygosity of 1.02%. Assembly with hifiasm yielded 1140 contigs totalling 1.15 Gbp with an N50 (i.e. 50% of the contigs were this length or longer) of 2.88 Mbp and N75 of 1.41 Mbp. The L50 (i.e. 50% of the total assembly length was contained in this many contigs) was 112 and the L75 was 256. The resulting *L. elliptica* assembly was shown to be highly complete with 97.1% of the 954 metazoan odb_10 BUSCOs detected as complete (90.3% of these were single-copy and 6.8% were duplicated) and another 0.6% detected as fragmented (table 1).

**Table 1.** Metrics of draft *Laternula elliptica* genome.

sequencing	
mean CCS read length	16 172 bp
CCS reads	1 389 345
total CCS bases	22 468 867 891
**genome assembly**	
assembly size	1 153 984 643
GC content (QUAST)	37.86%
coverage	19.47
contigs	1140
N50	2 884 873
N75	1 405 902
L50	112
L75	256
BUSCO (genome assembly)	97.1%[S:90.3%,D:6.8%],F:0.6%,M:2.3%,n:954
	926 complete BUSCOs (C)
	861 complete and single-copy BUSCOs (S)
	65 complete and duplicated BUSCOs (D)
	6 fragmented BUSCOs (F)
	22 missing BUSCOs (M)
	954 total BUSCO groups searched
**annotation**	
predicted transcripts	51 134
BUSCO (BRAKER annotation)	98.0%[S:86.7%,D:11.3%],F:1.5%,M:0.5%,n:954
	935 complete BUSCOs (C)
	827 complete and single-copy BUSCOs (S)
	108 complete and duplicated BUSCOs (D)
	14 fragmented BUSCOs (F)
	5 missing BUSCOs (M)
	954 total BUSCO groups searched

**Figure 2.**
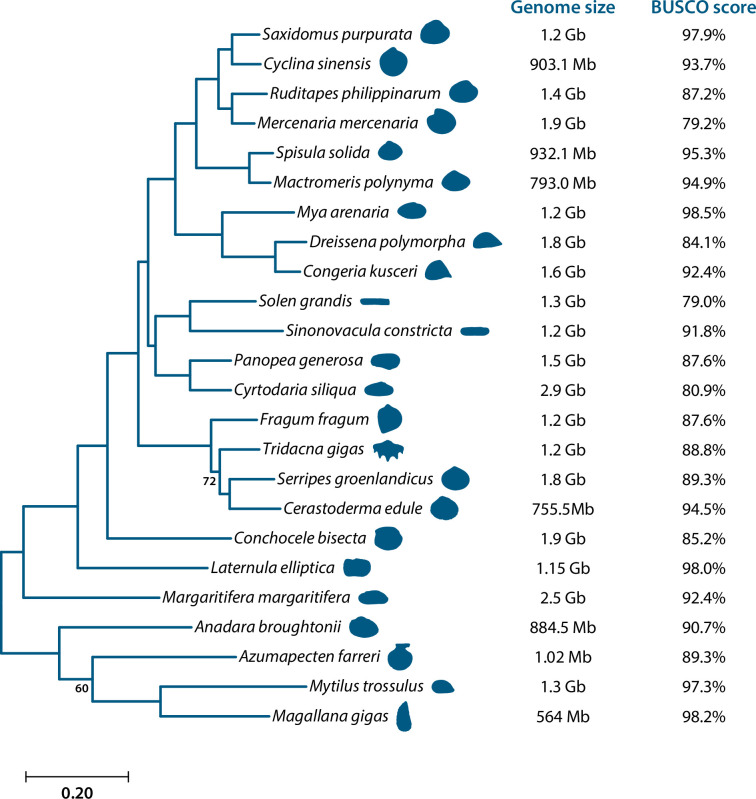
Phylogeny of bivalves using whole genome data. On the right of the tree, we provide associated genome size and BUSCO score for each species. For genome data associated with each species, see electronic supplementary material, S4.

Structural annotation in BRAKER yielded 51 134 predicted genes. Assessment of the completeness of the predicted genes revealed even higher values than that for the genome assembly with 98.2% of the 954 metazoan odb_10 BUSCOs detected as complete (86.9% of these were single-copy and 11.3% were duplicated) and another 1.3% were detected as fragmented (table 1; figure 2). The analysis of BUSCO scores based on predicted proteins from 23 other bivalve genome projects yielded scores from 79% in *Solen grandis* to 98.2% in *Magallana gigas* (figure 2 and electronic supplementary material, S1). While we did not achieve chromosome-level assembly with the *L. elliptica* genome, the data are highly complete, especially compared with other bivalve genomes, with only two other bivalve genomes (*Ma. gigas* and *Mya arenaria*) produced with over 98% completeness according to BUSCO scores (figure 2).

OrthoFinder identified 89 389 orthogroups (i.e. gene families) from 24 bivalve genomes (including *L. elliptica*), whose predicted proteomes had a BUSCO completeness score >80% (electronic supplementary material, S1). Our bioinformatic pipeline for tree reconstruction reduced this to 7126 alignments of orthologous sequences with an average of 20 taxa sampled per alignment. Concatenated, we produced a data matrix totalling 2 808 205 amino acids with 18.5% missing data. ML phylogenetic analysis of this matrix resulted in a strongly supported tree (figure 2) with maximal support for all nodes except one internal node within Cardiida and one internal node within Pteriomorpha. *Laternula elliptica* was recovered as the sister taxon of Imparidentia with maximal support, and higher level relationships within Imparidentia were consistent with the most recent phylogenomic analysis of the group [48] (figure 2). Estimation of gain/loss dynamics using CAFE5 inferred a birth–death parameter (*λ*) across the tree of 0.0017. After filtering the 89 389 gene families inferred by OrthoFinder, 1037 (out of 44 379) gene families were found to have undergone expansions or contractions in *L. elliptica.* Functional annotation with InterProScan resulted in 169 annotated gene families significantly (*p* < 0.05) expanded and 47 annotated gene families significantly contracted. Common expansions included the immune-associated C1q domain, elongation factors and chitin-binding domains. Contracted domains included G protein-coupled receptors (rhodopsin-like), epidermal growth factors (EGF-like) and others associated with cell fate, such as MAB21 (electronic supplementary material, S3).

### 3.2. RNA experiments showing age-dependent expression of shell damage response genes

**Figure 3.**
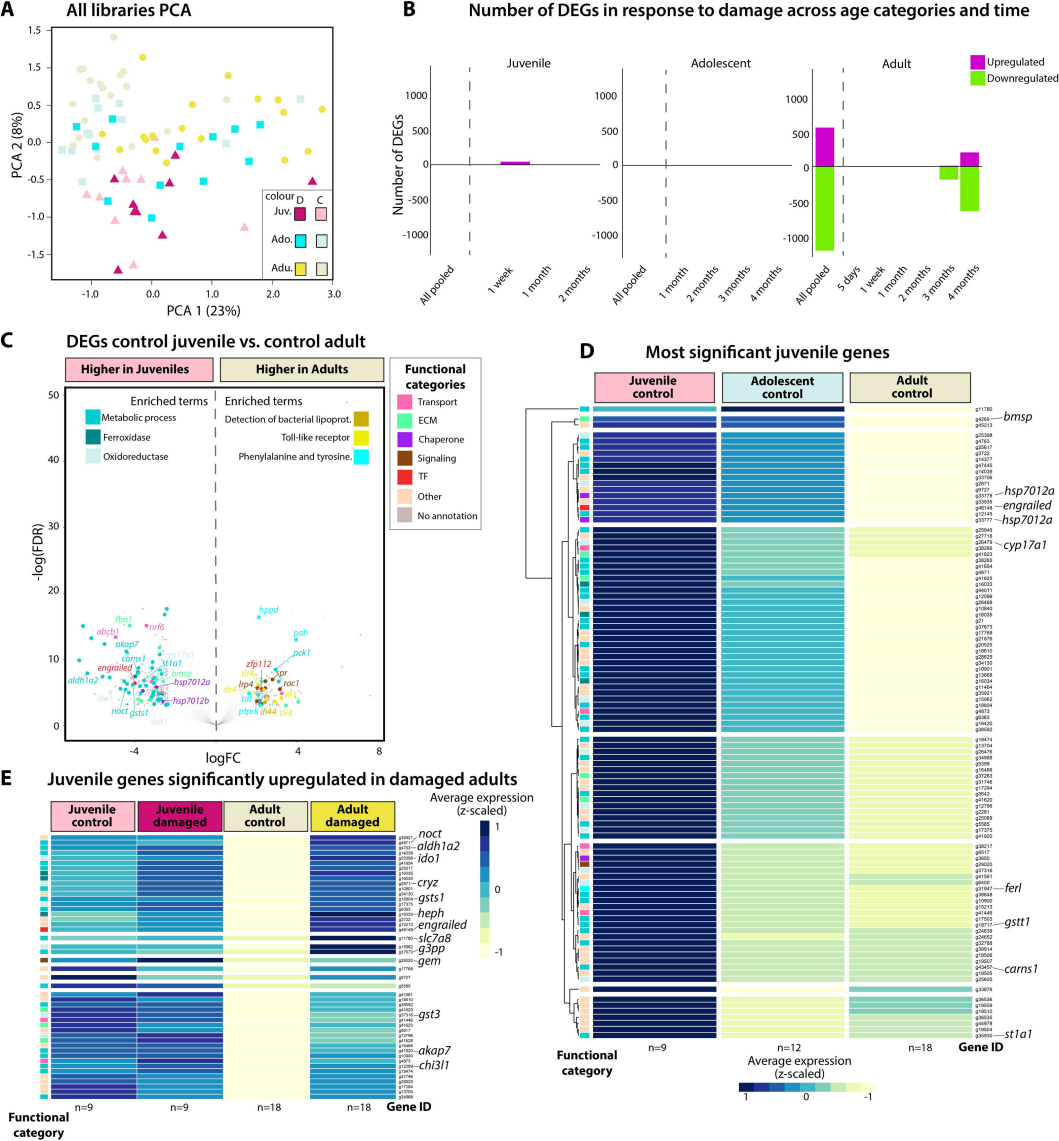
Transcriptomic experiment results. (A) PCA plot of all libraries. (B) Number of differentially expressed genes (DEGs) in response to damage across age categories and time. (C) DEGs in control animals: juveniles versus adults annotated by enriched molecular processes with annotation of associated genes. (D) Heatmap showing the 100 most significantly upregulated juvenile genes in control animals versus expression levels in adolescent and adult control animals. (E) The 100 most upregulated genes in juvenile control animals (as per D) which are upregulated in response to damage in adult animals. For reference, levels in damaged juvenile and control adults are shown.

The transcriptomics experiments (figure 1) aimed to understand the age-dependent damage repair response in this clam. Briefly, three age cohorts, juvenile, adolescent and adults, were subjected to shell damage experiments (figure 1C). The mantle tissue underlying the damaged shell areas was sampled over a time course (5 days to 4 months depending on age cohort) for RNA-seq, alongside a similar set of samples from control animals. These mantle RNA-seq data were previously analysed to produce the first computationally predicted gene regulatory network for molluscan biomineralization [47]. In the present study, we reanalysed the data to identify ‘age-specific’ genes, i.e. genes only expressed in juvenile or adult animals. These genes were then proposed as candidates as those whose expression is most likely controlled by methylation. The principal component analysis (PCA) plot of gene expression levels in the different age cohorts demonstrated the variable age effect, with no difference between control and damaged juvenile and adolescent animals. There was clear separation of the adults from the juvenile and adolescent animals (figure 3A). These data are corroborated by the number of DEGs identified in the different age cohorts in response to damage repair, with significant transcriptional responses to damage only found in adult animals (significance cut-off = FDR < 0.05) (figure 3B). To characterize the age-dependent transcriptional dynamics, we identified the DEGs of juvenile versus adult animals in control non-damaged tissues. These data were then subjected to enrichment analyses identifying the predominant functions and processes in both juvenile and adult animals (figure 3C). Most notably, in juvenile animals there was enrichment of the GO terms ‘Metabolic Process’, ‘Oxidoreductase’ and ‘Ferroxidase’. This list of genes that are more highly expressed in juvenile versus adult animals included genes with chaperone functions such as the heat shock proteins HSP70a12a and HSP702b and antioxidants including genes encoding superoxide dismutase and glutathione transferase. In contrast, in adult animals, the GO terms ‘Detection of Bacterial Lipoprotein’, ‘Phenylalanine and Tyrosine’ (genes including PAH (phenylalanine-4-hydroxylase), HPD (4-hydrophenylpyruvate dioxygenase) and TAT (tyrosine aminotransferase)) and ‘Toll-like Receptors’ (exemplified by the genes TLR1 and TLR4) were enriched (figure 3C). These data were mirrored in the STRING outputs (electronic supplementary material, S4). Interestingly, the STRING protein–protein interaction networks for both juvenile and adult animals centred on the ACTB (Actin, cytoplasmic 1) gene with other linked genes involved in signalling processes. However, in juvenile animals there were interactions of this hub with proteins involved in transcription/translation (e.g. elongation factor and eukaryotic initiation factor genes), detoxification (e.g. CRYZ (quinone oxidoreductase) and NQO2 (ribosyldihydronictotinamide dehydrogenase)) and transport (e.g. acetyl choline receptor genes). In adult animals, the STRING network was more extensive. The interactions of the ACTB hub were largely with proteins involved in metabolism (e.g. genes such as PAH, TAT, PCK1 (phosphoenolpyruvate carboxykinase)) and glyoxylate reductase and the immune response (e.g. TLR genes) (electronic supplementary material, S4). The genes most significantly expressed at higher levels in juvenile compared with adult tissue were plotted as a heatmap, including the intermediate adolescent stage, confirming a general pattern of decreasing expression with age (figure 3D).

To identify genes that were potentially methylated with age to reduce or shut down specific functions, we focused our analyses on the 100 most highly expressed annotated genes in juvenile control animals that significantly decreased in expression levels in adolescent and adult control animals (referred to as juvenile genes hereafter) (figure 3D; electronic supplementary material, S2). Of these 100 juvenile genes, none significantly responded to damage in juvenile animals (figure 3E). These data included genes with very different functions, involved in processes including the cytoskeleton (actin and tubulin genes), ubiquitylation (E3 SUMO protein ligases), antioxidants including several members of the glutathione synthase family and the atypical HSP70 family chaperones HSP7012a and HSP7012b. None of the 100 juvenile genes were expressed in the mantle of non-damaged adult animals (controls), but 47 of these juvenile genes were upregulated (i.e. switched back on) in adult animals in response to shell damage (figure 3E). Interestingly, none of the cytoskeleton or HSP70 genes were reactivated, despite the potential cellular stresses arising due to shell damage. There was a trend for more of the multifunctional proteins with oxidoreductase and biosynthetic activities (e.g. quinone oxidoreductase, ribosyl dihydronicotinamide dehydrogenase and glycerol-3-phosphate phosphatase) to be upregulated in the adult animals in response to shell damage, along with transcription factors and signalling molecules (engrailed and notch), which have been previously implicated in shell formation (figure 3E electronic supplementary material, S2).

### 3.3. Methylation analysis showing lack of methylation associated with age-dependent shell damage response genes

We then analysed the methylation status of the juvenile genes to identify if any were differentially methylated and therefore subject to epigenetic regulation.

For this analysis, five juvenile clams and five adult clams were subjected to reduced representation EM-seq™ (Enzymic Methyl-seq). This was to identify methylated regions of the genome in juvenile and adult animals and whether these correlated with (and potentially offered a causative mechanism for) the DGE patterns observed in the transcriptomics experiment. In total, 989 485 766 reads were generated from the 10 age-specific samples and the mixed control (electronic supplementary material, S5). Similar numbers of reads were mapped in juvenile (201 052 391 reads) and adult (201 052 391 reads) animals. Although many CpGs were covered by too few reads (cut-off used was at least 20 reads in at least 2 juvenile and 2 adult samples; for more details see Material and methods) to allow a direct comparison between juvenile and adult samples, there were still tens of thousands of CpGs for analysis (CpGs with >20 reads mapped in all animals, total 12 345 or 73% of total differentially expressed genes; electronic supplementary material, S5). Methylation levels were higher in exons than the whole genome and higher in exons than introns. A *t*‐test was used to compare average methylation levels between juvenile and adult samples. Adult samples were found to be significantly more methylated than juvenile samples across the whole genome (*p* = 0.024), across introns (*p* = 0.008) and across exons (*p* = 0.015) (electronic supplementary material, S5). However, the situation was complex. There were few individual CpG regions statistically significantly differentially methylated with age across the genome with age (141 sites with an FDR < 0.05). Of these sites, only 6 exonic and 24 intronic sites were significantly more methylated in adult animals compared with juveniles, with 7 exonic and 26 intronic sites significantly more methylated in juvenile animals compared with adult animals (electronic supplementary material, S5). None of the differentially methylated sites corresponded to the genes identified as being significantly differentially expressed in our age-dependent transcriptomic analyses. Hence, methylation was unlikely to have impacted their transcription status and age-dependent expression patterns.

## 4. Discussion

The data presented here have provided a new understanding of age-dependent transcriptional dynamics in the relatively long-lived Antarctic clam *L. elliptica.* The reactivation of many ‘juvenile’ genes in adult animals upon damage demonstrated the temporary nature of the regulatory state of these genes, suggesting an epigenetic mechanism for transcriptional regulation. However, our data showed that epigenetic regulation via methylation was highly unlikely and suggested that other, as yet unidentified, epigenetic mechanisms are involved in the regulation of physiological maturity and ageing processes in *L. elliptica*.

Evaluation of methylation (and other epigenetic) data is most effectively achieved by mapping the results against a genome. Despite the dramatic improvements in genome sequencing in recent years, the extraction of high-quality nucleic acids from molluscs and production of high-quality contiguous genomes is still problematic [49]. Although the highly contiguous assembly produced here is more than adequate for the analyses we sought to carry out in this research, we originally aimed to generate a chromosome-level genome assembly for *L. elliptica* with the addition of Hi-C chromatin conformation data (for reference, the chromosome complement of *L. elliptica* is 2*n* = 40 [50]). However, attempts in the Kocot laboratory and by Phase Genomics to produce Phase Genomics Animal Hi-C libraries consistently failed. We experienced similar difficulties in producing Hi-C libraries from the bivalve *Aequiyoldia eightsii* (Jay, 1839), whereas library preparation from less ‘slimy’ molluscs including the scaphopod *Siphonodentalium dalli* (Pilsbry & Sharp, 1898) and two solenogaster aplacophorans worked well (data not shown), suggesting mucus could play a role in the challenges we faced, as has been identified in other mollusc species [49,51]. Despite these technical problems, the *L. elliptica* genome is highly complete, with only two other bivalve genomes (*Ma. gigas* and *My. arenaria*) produced with over 98% completeness according to the BUSCO scores (figure 2).

Within the Mollusca, bivalves tend to have moderate *C*-values [52]. This is typified by *L. elliptica* with a draft genome size of approximately 1.15 Gb, which is intermediate in size compared with the other bivalve genomes used in the phylogenetic tree (figure 2). In keeping with the *C*-value paradox, the closest bivalve to *L. elliptica* in the phylogeny is *Conchocele bisecta,* which has the largest genome (1.9 Gb) of the species used in the phylogenetic analysis. However, this species is rather unusual compared with the others in that it inhabits deep sea hydrothermal vents. It obtains nutrients through chemosymbiosis and therefore the genome is a hologenome, with particular adaptations to this chemosynthetic mode of metabolism [53].

Genome analysis revealed 1037 gene families with significant expansions or contractions in *L. elliptica* compared with other bivalves, of which 167 were annotated using Pfam and InterPro (electronic supplementary material, S3). The most expanded domains in *L. elliptica* included those involved in functions such as cell adhesion and cell interactions (e.g. GTPase and vWA-like domains), signalling processes (EGF and EGF-like) and immune functioning (C1q, lectin C-type and thrombospondin). While many of the latter are hallmarks of the invertebrate innate immune response, these domains can also be multifunctional (e.g. c-lectin, wVA and EGF-like) and in particular have been identified in biomineralization [54]. Biomineralization is important in *L. elliptica* as this species has a very thick shell, presumably due to living in a habitat where being hit by an iceberg is a frequent risk, and indeed many *L. elliptica* shells show extensive damage repair as a result [55]. Thus, expansion of these domains could be driven by the requirement for a robust shell and efficient shell repair processes.

Another driver for this loss and gain of gene families is likely to be adaptation to the cold, as exemplified by the numerous expansions and contractions of gene families in Antarctic fish genomes [56]. These ‘extra’ genes in notothenioid (Nototheniidae) genomes, which include antifreeze glycoproteins and genes involved in ROS homeostasis are a likely result of adaptation to life in freezing waters [57,58]. Some expansions may also be due to the problems of making proteins in the cold, where one evolutionary solution that is prevalent in Antarctic fish is not evolution of the amino acid coding sequence of the protein to work better in the cold but merely to produce more of the protein (with the same amino acid sequence as temperate congeners) via duplicated genes [59]. This may explain the expansion of, for example, the immune genes in *L. elliptica.* These genes are particularly important in bivalves that have a sessile benthic lifestyle, perpetually surrounded by and ingesting sea water with a very high microbial content and if the immune proteins do not work well in the cold, then expansion of the immune gene repertoire may be needed in *L. elliptica.* There are very few Antarctic invertebrate genomes sequenced, but even the compact 145 Mb genome of the Antarctic winged midge *Parachlous steinenii* showed a significant number of expanded and contracted gene families (806 and 2567, respectively). Of these, 65 gene families were subject to rapid evolution and included genes involved in cold adaptation such as acyl-CoA delta desaturase and HSP70 [60]. Thus, the expanded/contracted gene families in *L. elliptica* are likely the result of habitat-specific drivers, in which ice, temperature and biomineralization are major players. It should be noted that the prevalence of reverse transcriptase and Harbinger repeat element domains in both the expanded and contracted gene families in *L. elliptica* is likely due to uncharacterized repeat elements that were not identified or masked by our repetitive DNA annotation and masking strategy, especially as Harbinger elements lack transposase activity [61].

Working with non-model species can be challenging when transferring analytical technologies, such as epigenetic screening protocols from more traditional model species, such as humans and mice. In this respect, the *L. elliptica* genome here provides a critical tool for further epigenetic investigations and dissection of age-dependent responses. A conventional transcriptional analysis of damage-repair expression profiles across the three age cohorts revealed no significant results in juvenile and adolescent animals, with a significant number of damage-repair-related DEGs identified in adult animals after three to four months (figure 3B). In addition, there was a clear difference in transcriptional profile between control animals with age (figure 3C,D).

Such age-dependent transcriptional profiles have previously been identified in *L. elliptica* in response to environmental challenges, such as injury, starvation, hypoxia and warming [2,17,18]. However, these studies were carried out with less resolution (single time point experiments), compared with the current study. Our use of two young age cohorts in comparison to adult animals over a time course experiment and the evaluation of control and treated animals enabled the discovery of a clear delineation in expression profiles between non-reproductive (immature juveniles and adolescents) and reproductively active (adult) animals. These molecular data correspond to previous biochemical analyses and physiological parameters investigated in a range of bivalve molluscs, most notably the very long-lived *Arctica islandica* [62]. Investigation of biochemical markers across *A. islandica* individuals ranging in age from 4 to 192 years demonstrated a rapid reduction in the activities of catalase and citrate synthase and concentrations of glutathione in the first 25 years of life, followed by stable levels for the following 150 years. This initial decline was associated with rapid somatic growth and the onset of sexual reproduction followed by the stabilization of cellular biochemistry at maturity [62]. Shell growth data in other molluscs show a similar pattern, with rapid early growth, followed by an exponential reduction in growth rate as the maximum size of the species is approached. This phenomenon is accurately modelled using the Von Bertalanffy equation [63] and has been clearly demonstrated in both *L. elliptica* and another relatively long-lived Antarctic bivalve, *Aequiyoldia eightsi* [64,65]. Furthermore, there are secondary age effects in these species, where *A. eightsi* >27 mm did not grow through the winter, but animals <27 mm grew at the same rate throughout the year [66] and in *L. elliptica* age is decoupled from animal size in older animals, even though markers of ageing such as accumulation of lipofusan increase with age [11,23]. This age-dependent pattern is thought to be due to a trade-off between somatic growth and resource allocation to reproduction [66] and conform to the *A. islandica* data.

Such age-dependent traits are more prominent in *L. elliptica* because this is a relatively long-lived species. However, the promotion of this species as an ageing model has been largely superseded by the study of much longer lived, and more easily available bivalves, such as *A. islandica* and *Margaritifera margaritifera*, with maximum recorded life spans of 507 and 190 years, respectively [7]. The relatively long life span of *L. elliptica* compared with the majority of temperate and tropical bivalves can be explained by the slowing of physiological parameters with temperature in cold-adapted species coupled with long bouts of metabolic depression during the Antarctic winter [67–69]. The slowing of biological processes in the cold also includes delaying age of reproductive maturity, which is positively correlated with longevity [70]. The delayed reproductive maturity and the associated lack of reproductive senility with age in molluscs [70] fit the same pattern as the shell growth data, discussed above, with stabilization of metabolic processes at maturity.

Overall, there is a coherent pattern of bipartite traits in *L. elliptica*, demonstrating one set of cellular, physiological and morphological processes associated with rapid early growth, followed by stabilization of these processes at maturity, which is shared with other bivalve molluscs. The juvenile/adult transcriptional profiles in *L. elliptica* are, therefore, likely due to the physiological changes associated with sexual maturity and not, as is often assumed, due to senescent loss of these capacities [2,12,62]. However, the genes that trigger this switch in physiology from pre- to post-reproductive maturity are still unknown, as is the mechanism by which many different biochemical pathways are ‘silenced’ or regulated at maturity. A prime example of this phenomenon in our damage-repair transcriptional data is the ‘reactivation’ in adult damaged animals of signalling molecules such as *engrailed* and *notch*. These genes are frequently associated with developmental processes but also cell differentiation, proliferation, apoptosis and shell formation [71,72]. In adult molluscs, new shell growth occurs at a very low level (as described above), but shell damage requires at least a temporary elevation in gene expression of shell formation pathways to sustain shell growth and repair [72]. A key player in gene regulation is epigenetics. One of the main aims of our study was to identify juvenile genes that were inactivated with age and therefore were considered prime targets for epigenetic regulation via methylation.

Epigenetic mechanisms can take several forms, most commonly, involving methylation of cytosines, histone modification and/or non-coding RNAs. To date, the vast majority of epigenetic studies in molluscs investigated methylation patterns and their association with gene regulation [73]. These studies have provided ample evidence that molluscs possess the required DNA methyl transferases to effect methylation of cytosines and that such methylation is an active epigenetic modifier of transcription in this taxon [51,73]. There are very few studies (and associated transfer of technologies) investigating the role of histone modifications and non-coding RNAs in mollusc epigenetics. Therefore, in our study, the obvious starting point for the evaluation of epigenetic control of ageing transcriptomes was methylation, which we chose to evaluate using EM-seq.

Our data strongly aligned with those of other invertebrate and mollusc studies in showing substantial levels of cytosine methylation of CpGs in the *L. elliptica* genome, the majority of which was intragenic [51,73,74] (electronic supplementary material, S5). In this respect, invertebrate epigenetic mechanisms fundamentally differ from those of vertebrates, which have higher levels of CpG methylation that is distributed globally [75,76]. There was significantly more methylation of CpGs in adult animals; however, the number of significantly differentially methylated genes was very low across the whole genome, with none associated with the age-dependent genes in our study, suggesting that methylation was not a significant factor in the age-dependent gene expression results. Previous studies in molluscs, particularly the genus *Crassostrea*, have shown that methylation of cytosines is a key feature in developmental regulation [77,78], but these studies concentrated on larval development and not the later pre- and post-reproductive stages examined here. A more comprehensive study using the chemical Vinclozolin to disrupt methylation in juvenile and adult freshwater snails (*Physella acuta*) showed a reduction in methylation with age [79]. Nonetheless, similar to the *Crassostrea* studies, the dramatic decline in methylation levels in *P. acuta* occurred in the first 50 d when the animals were still developing and highly immature [79,80]. Around the boundary age for maturity, and after reproductive maturity, there was very little change in methylation levels in *P. acuta*, mirroring the results in the present study.

Although there are data from other species suggesting that epigenetics plays a role in ageing [21,22], our data strongly suggest that methylation of cytosines is not the mechanism active in the ageing process of the Antarctic clam *L. elliptica*. There is clearly some mechanism operating to maintain a steady physiological state post reproductive maturity, which takes time to reverse in this clam, if the genes under control need to be reactivated in response to an environmental challenge. This is more complex than simplistic transcription of a gene in response to an environmental trigger, with some type of epigenetic control hypothesized. For example, a previous study that correlated methylation levels with acclimation to the intertidal in the Antarctic limpet *Nacella concinna* included a common garden experiment, where the methylation markers took more than nine months to erase [81]. Furthermore, a recent study of epigenetics in the purple sea urchin *Stronglycentrotus purpuratus* indicated that methylation alone was insufficient to control transcription and that transcription was affected by interactions with genic architecture and changes in chromatin access [82]. Although significant levels of methylation were identified in the *L. elliptica* data presented here, from a more global perspective, molluscs are considered to be hypomethylated compared with other members of the tree of life [83]. Clearly, more investigation is needed to understand the mechanisms for transcriptional regulation and maintenance of physiological state in molluscs. In this respect, histone variants represent prime, and as yet unexplored, candidates in the Mollusca [84].

In summary, the generation of the first draft genome of the Antarctic clam *L. elliptica* provides valuable information on evolution to infaunal life in very cold waters, with the expansion of gene families potentially involved in the immune response and biomineralization. The genome also provides a resource for the detailed molecular investigation of this species, in particular, the age-dependent environmental response. The damage-repair data described here clearly demonstrate a bipartite molecular homeostasis in *L. elliptica,* which is associated with a rapid growth phase in pre-reproductive juveniles and a stabilization in adults post-reproductive maturity. The trigger for this change in physiological state is, as yet, unknown, but we can now rule out methylation as an epigenetic mechanism as the sole cause of this process. The slowing of biological processes in *L. elliptica* provides additional granularity for investigating the underpinning molecular mechanisms that are affected by ageing. The new *L. elliptica* genomic tools described here provide a real opportunity for further exploitation of the different epigenetic factors acting in this species, which are likely applicable to a wide range of molluscs.

## Data Availability

Genome data: the *Laternula elliptica* HiFi reads and genome assembly: NCBI BioProject accession number PRJNA875466. The Whole Genome Shotgun project: accession number JBKBCU000000000. EM-seq data: ENA BioProject accession number PRJEB73630. Genome annotation and phylogeny data: the genome assembly (with and without softmasking), gene models (including predicted coding sequences, protein sequences and gff3 file), InterProScan functional annotations and files related to the GenomeScope analysis, phylogenetic analysis, gene gain/loss analysis and differential gene expression analysis. Figshare [85]. Electronic supplementary material is available online [86].
